# Effects of different delivery forms of the novel *Bacillus subtilis* strain 87Y on redox status and liver function in peri-weaning piglets

**DOI:** 10.2478/jvetres-2026-0051

**Published:** 2026-09-16

**Authors:** Anna Czech, Łukasz Wlazło, Marcin Łukaszewicz, Agnieszka Lewińska, Wojciech Łopuszyński, Bożena Nowakowicz-Dębek

**Affiliations:** 1 Department of Animal Hygiene and Environmental Hazards, Department of Biochemistry and Toxicology, Faculty of Animal Sciences and Bioeconomy, University of Life Sciences in Lublin, 20-950 Lublin, Poland; 2 Department of Biochemistry and Toxicology, Faculty of Animal Sciences and Bioeconomy, University of Life Sciences in Lublin, 20-950 Lublin, Poland; 3 Department of Biotransformation, Faculty of Biotechnology, Faculty of Chemistry, University of Wrocław, 50-383 Wrocław, Poland; 4 Faculty of Chemistry, University of Wrocław, 50-383 Wrocław, Poland; 5 Department of Pathomorphology and Forensic Veterinary Medicine, Faculty of Veterinary Medicine, University of Life Sciences in Lublin, 20-612 Lublin, Poland

**Keywords:** fermented rapeseed meal, *Bacillus subtilis* 87Y, ferric-reducing antioxidant power, solid-state fermentation, weanling pigs, zinc oxide

## Abstract

**Introduction:**

Weaning is a critical stage in piglet rearing, and dietary strategies are needed to support metabolic balance and oxidative resilience during the peri-weaning period.

**Material and Methods:**

A total of 384 Yorkshire × Danish Landrace piglets at 28 days old were allocated to four groups: positive controls with feed additives including ZnO (PC), negative controls without additives (NC), NC + 8% fermented rapeseed meal (FR) and NC + 0.1% *Bacillus subtilis* 87Y (BS). Blood was collected from 24 males (one per replicate) for redox and biochemical analyses, and liver and small intestine samples were collected *post mortem* for oxidative marker assessment and histopathology.

**Results:**

Compared with NC, FR and BS supplementation increased antioxidant defence (catalase, superoxide dismutase and glutathione) and reduced lipid peroxidation markers (malondialdehyde and lipid hydroperoxides) in plasma and tissues; changes were accompanied by favourable shifts in selected lipid metabolism indices and liver enzyme activities. The pure probiotic form of *Bacillus subtilis* was not inferior to the fermented matrix and, for selected parameters, produced an effect stronger than that observed in the PC diet.

**Conclusion:**

*Bacillus subtilis* 87Y improved redox status and supported liver function in peri-weaning piglets, with efficacy largely independent of the delivery form, indicating potential as a feed additive.

## Introduction

Weaning is one of the most critical stages in piglet rearing, as it involves abrupt environmental, nutritional and social changes accompanied by a substantial physiological burden (18, 29). The sudden transition from sow’s milk to solid feed induces multifactorial stress, leading to intensified catabolic processes, gut microbiota dysbiosis, reduced immune competence and disturbances in metabolic functions (29). An additional factor predisposing piglets to impaired homeostasis is the immaturity of their physiological systems, which contributes to increased production of reactive oxygen species (ROS) and the development of oxidative stress, regarded as one of the key health threats during this period (6, 16, 21). Under physiological conditions, the balance between ROS generation and the activity of enzymatic and non-enzymatic antioxidant systems is tightly regulated. In piglets, however, these mechanisms are insufficiently developed, limiting effective neutralisation of free radicals. Consequently, lipid peroxidation is intensified, resulting in damage to membrane structures, increased intestinal barrier permeability, activation of inflammatory responses and deterioration of metabolic and immunological indicators (16). The liver is particularly susceptible to oxidative injury, as it is the central organ for metabolism and detoxification (3).

In recent years, alongside increasing recognition of the importance of oxidative and immune stress during the peri-weaning period, there has been a clear need to identify alternatives to the commonly used zinc oxide. Its use has been substantially restricted in the European Union because of environmental concerns and the risk of promoting bacterial resistance to antibiotics (25). As a result, interest has grown in feed additives that could effectively support gut health, immunity and redox balance in young pigs while maintaining a high safety profile (19). In this context, *Bacillus* probiotics are of particular interest because of their endospore-forming ability, which confers high stability and survivability in the gastrointestinal tract (5).

A growing body of evidence indicates that these probiotics modulate the composition and activity of the gut microbiota, improve intestinal epithelial morphology, reduce pathogen translocation, stimulate immune responses, and influence host redox status through both direct mechanisms (*e.g*. production of antioxidant enzymes, bioactive peptides and reducing compounds) and indirect mechanisms (*e.g*. stabilisation of gut function and attenuation of inflammation) (1, 29, 30). Accordingly, *Bacillus subtilis* emerges as a promising alternative to ZnO, potentially capable of mitigating the adverse consequences of peri-weaning stress, improving metabolic function and supporting intestinal barrier integrity.

However, the efficacy of *Bacillus* probiotics is strongly determined by strain-specific properties and by the mode of administration. A crucial role is played by the delivery matrix, which affects microbial survival in the gastrointestinal tract, metabolism and interactions with the host. The matrix may act as an inert carrier or may additionally provide bioactive substances that act synergistically with the probiotic (14). Therefore, it is fully justified to combine comparative study of matrix benefits with investigation of novel probiotic strains with antioxidant and immunomodulatory potential.

The aim of the present experiment was to comprehensively evaluate the efficacy of a novel *Bacillus subtilis* 87Y strain not previously applied in pig nutrition, when administered in different matrices (spores *versus* a fermented carrier). The study was conducted in a piglet model and included assessment of redox status indices in plasma and hepatic and intestinal tissues, determination of biochemical parameters and lipid profile, as well as histological evaluation of the liver and kidneys. The findings will help to determine the suitability of this strain as an innovative feed additive supporting piglet health and development during the stress-sensitive rearing period and help to clarify the influence of the administration form on its biological efficacy.

## Material and Methods

All experimental procedures were approved by the Local Ethics Committee for Animal Experimentation in Lublin, Poland (approval No. 50/2018, April 1, 2018).

### Preparation of fermented rapeseed meal (FRSM)

Fermented rapeseed meal was produced by solid-state fermentation using *Bacillus subtilis* strain 87Y. Rapeseed meal was sterilised at 121°C for 10 min to eliminate microbial contamination. The strain was cultured overnight in lysogeny broth at 37°C until an optical density of approximately 0.1 at 600 nm was reached. The resulting culture was mixed with the sterilised rapeseed meal and the final moisture content was adjusted to 50% (w/w). Fermentation was carried out at 37°C for 24 h under aerobic conditions with periodic aeration. After fermentation, the material was dried, milled and stored until further use.

### Bacterial strain

The *B. subtilis* 87Y strain was originally isolated from the earthworm *Eisenia fetida* (28). Culture preparation was performed according to the method described by Wlazło *et al*. (32). Solid-state fermentation was conducted in a modular bioreactor consisting of six independent modules with a total working capacity of up to 5 m^3^. The inoculum was prepared in a 470 L (total volume) liquid fermenter (Boccard-Kates, Olsztyn, Poland).

### Animals and experimental design

A total of 384 Yorkshire × Danish Landrace piglets were used, comprising 192 barrows and 192 gilts. The experimental period started immediately after weaning at 28 days of age and continued until the piglets reached a final body weight of approximately 30 kg. Throughout the nursery period, animals had *ad libitum* access to feed and water. However, the experimental diets were introduced earlier, while the piglets were still with the sows, to facilitate adaptation to new feed sources and to support the weaning process.

After transfer to the nursery facility, all piglets were individually identified, weighed and randomly allocated to one of four dietary groups, with an equal sex distribution in each group. Each group comprised six pen replicates, with 16 piglets per pen (8 gilts and 8 barrows). The mean initial body weight ranged from 9.56 ± 0.799 kg in the unsupplemented standard-diet group to 9.70 ± 0.917 kg in the supplemented standard-diet group, while in the fermented-rapeseed-and *Bacillus subtilis*-supplemented groups it was 9.62 ± 0.944 kg and 9.66 ± 1.07 kg, respectively.

A schematic overview of the experimental design, dietary treatments, sampling procedures and analytical workflow is presented in Fig. 1.

**Fig. 1.**
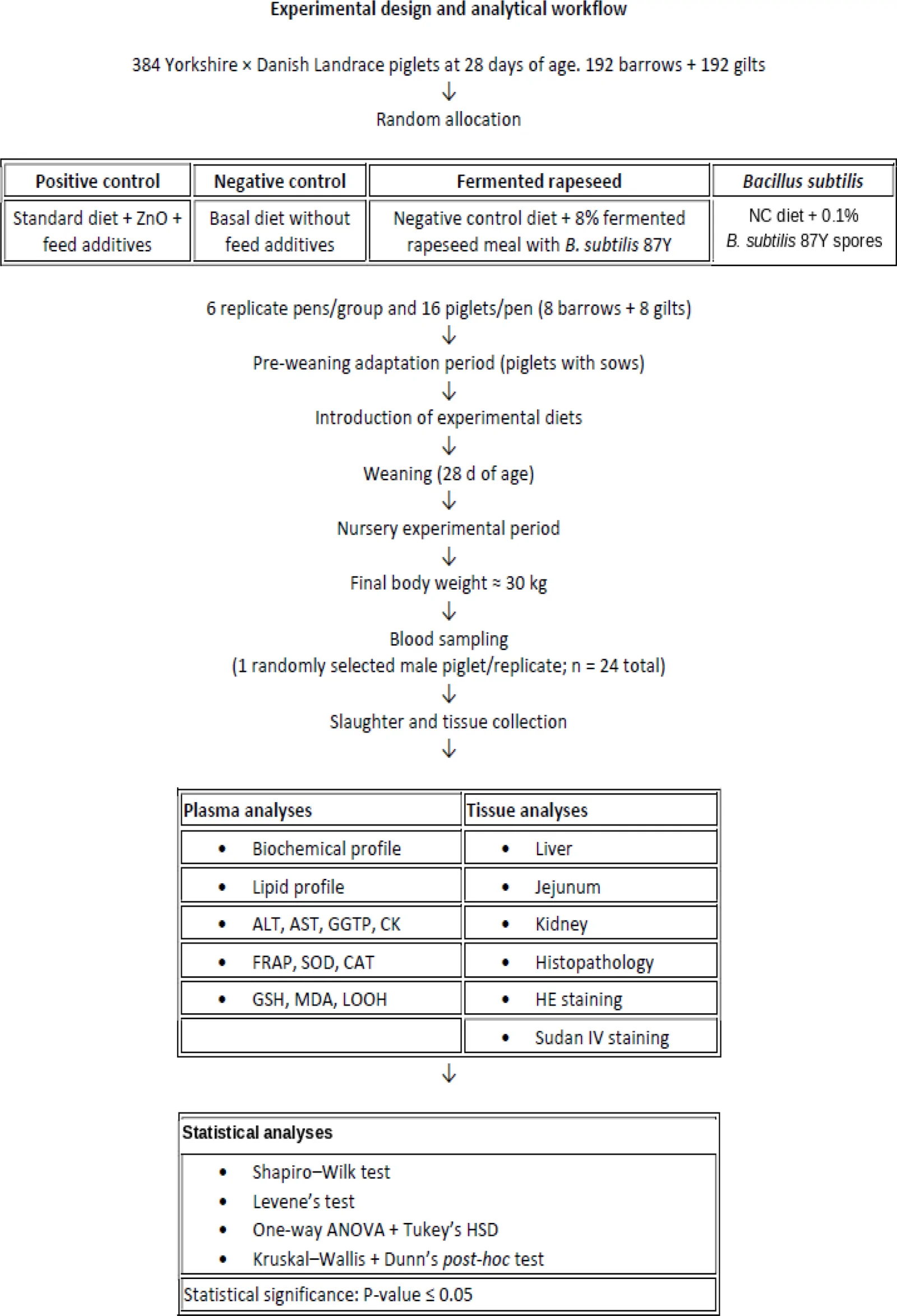
Schematic overview of the experimental design, dietary treatments, sampling procedures and analytical workflow used to evaluate the effects of *Bacillus subtilis 87Y* administered in different delivery forms in peri-weaning piglets. ZnO – zinc oxide; NC – negative control; B. subtilis – Bacillus subtilis; ALT – alanine aminotransferase; AST – aspartate aminotransferase; GGTP – γ-glutamyltransferase; CK – creatine kinase; FRAP – ferric-reducing antioxidant power; SOD – superoxide dismutase; CAT – catalase; GSH – glutathione; MDA – malondialdehyde; LOOH – lipid hydroperoxides; HE – haematoxylin and eosin; ANOVA – analysis of variance; HSD – honestly significant difference

### Experimental diets

Four experimental diets were used. The positive control group (PC) received a standard diet appropriate for the growth stage, supplemented with enzymes, acidifiers, ZnO, probiotics, prebiotics and antioxidants. The negative control group (NC) was fed a diet identical to the PC group’s but without feed additives. In the FR group, the diet was based on the NC formulation and supplemented with 8% FRSM, which replaced part of the soybean meal. In the BS group, the diet was also based on the NC formulation and supplemented with 0.1% of the novel probiotic strain *Bacillus subtilis* 87Y. All diets were formulated according to US National Research Council’s recommendations (24) for nutrient content, except for in respect of zinc, the level of which in the NC, FR and BS diets was unmodified from the standard level in the feed ingredients.

The dietary formulations were balanced to provide comparable crude protein (approximately 16.2%) and crude fibre (approximately 3.4% in the PC, NC and BS diets; and 3.84% in the FR provision), and their crude fat contents ranged from 3.2% in PC and BS to 3.78–3.87% in FR and NC. The diets included cereals, soybean meal, full-fat soybean, fish meal, soybean oil and mineral and vitamin supplements. The contents of essential amino acids (lysine, threonine and methionine) and of calcium and phosphorus were adjusted to maintain equivalent nutritional value across all diets. All mixtures contained 3 g/kg of a mineral and vitamin premix providing vitamins A (11,250 IU), D_3_ (2,250 IU), E (90 mg, including 81.81 mg DL-α-tocopherol; 33.52 IU biologically active), K (2.25 mg), B_1_ (2.25 mg), B₂ (5.64 mg), B_6_ (3.39 mg), B_12_ (0.027 mg), pantothenic acid (12 mg), niacin (22.5 mg), biotin (0.113 mg), choline (500 mg) and folic acid (0.90 mg), as well as minerals, namely Fe (112.5 mg), Cu (150 mg), Mn (78.75 mg) and Se (0.45 mg). In addition, the PC group received 3.5 g/kg of feed additive to optimise nutrient utilisation and support gut health. This comprised ZnO (0.3% DM), organic acids (0.3% DM), phytase (0.005% DM), protease, endo-β-glucanase and endo-β-xylanase (0.01% DM) and *Saccharomyces cerevisiae* (0.01%).

Throughout the experiment, piglets had *ad libitum* access to feed and water. Before the start of the trial, all animals were examined by a veterinarian to confirm good health status. Mortality was low and did not differ among groups.

### Experimental procedures

At the end of the experiment, when piglets weighed approximately 30 kg, blood samples were collected from one randomly selected male piglet from each replicate to determine redox parameters and plasma biochemical indices (4 groups × 6 males = 24 animals). The animals were fasted for 12 h before blood collection. Blood was drawn from the anterior *vena cava* using a sterile 22-gauge needle. Samples were aliquoted into tubes containing heparin. Plasma was separated from heparinised blood by centrifugation for 10 min at 3,000 × *g* and stored at –80°C until analysis. On the following day, the animals from which blood had been collected were removed from their pens. These piglets were humanely euthanised by exsanguination after prior stunning with a penetrating captive bolt, in accordance with Council Regulation (EC) No. 1099/2009 (8). Immediately after euthanasia, dissection was performed; the heart, liver and kidneys were weighed, and samples of the liver, kidney and small intestinal wall were collected for analyses.

### Analytical procedures

Plasma concentrations of glucose (Glu), total protein (TP), albumin (ALB) and globulins (GLOB) were determined, and the ALB : GLOB ratio was calculated. In addition, plasma concentrations of urea, creatinine (CREAT) and bilirubin (BIL) were measured. The activities of selected liver enzymes, including ALT, AST, LDH, CK, and γ-glutamyltransferase (GGTP), were determined spectrophotometrically using an automatic biochemical analyser (Pentra C400; Horiba, Kyoto, Japan). Plasma lipid indices were also assessed, including total cholesterol (CHOL), high-density lipoprotein cholesterol (HDL) and triacylglycerols (TG). Low-density lipoprotein cholesterol (LDL), HDL percentage and the HDL : CHOL ratio were calculated.

Liver and small intestinal wall tissue homogenates were prepared from freshly collected samples, which were homogenised under refrigerated conditions (4°C). For the determination of GSH and MDA, 1 M HClO_4_ containing 2 mM EDTA was used, and for the determination of SOD and CAT, Sörensen phosphate buffer (pH 6.4) and phosphate buffer (K_2_HPO_4_/KH_2_PO_4_, pH 7.0) were used, respectively. Homogenisation was performed at a ratio of 1 : 4 (w/v) using a rotor-stator homogeniser. The samples were then frozen and stored until analysis. Before the assays, the homogenates were thawed and centrifuged at 6,000 rpm for 10 min at 4°C, and the resulting supernatants were used for analysis. All results obtained for tissue homogenates were expressed per 1 g of wet tissue.

In tissue homogenates and blood plasma, the concentration of malondialdehyde (MDA) was determined using the TBARS method, based on the reaction of MDA with thiobarbituric acid under acidic conditions and elevated temperature, with absorbance measured at 532 nm (26). Lipid peroxidation products: lipid hydroperoxide (LOOH) concentration was determined according to Gay and Gębicki (15). The concentration of reduced glutathione (GSH) was determined using Ellman’s reagent (DTNB), based on the reaction of thiol groups with 5,5′ -dithiobis-(2-nitrobenzoic acid), with absorbance read at 412 nm (11). Superoxide dismutase activity was determined based on the inhibition of adrenaline auto-oxidation monitored spectrophotometrically at 320 nm (17). Catalase activity was assessed by measuring the rate of enzymatic decomposition of H_2_O_2_ recorded as a decrease in absorbance at 240 nm (7). Ferric-reducing antioxidant power (FRAP) was determined only in plasma using a method based on measuring the ability to reduce the Fe^3+^-TPTZ (2,4,6-tripyridyl-s-triazine) complex to Fe^2+^-TPTZ at 593 nm (4). All determinations were performed spectrophotometrically in duplicate according to widely used procedures described in the literature (11).

### Histopathological analysis

Four 0.5-cm-thick liver samples from the right lateral, right medial, left medial and left lateral lobe, and one sample from each kidney were taken for histopathological examination immediately after euthanasia and fixed for 24 h in 10% buffered formalin (pH 7.2). The samples were then processed in graded concentrations of ethanol and xylene in a tissue processor (TP-1050; Leica Biosystems, Nussloch, Germany) and embedded in paraffin blocks. Histological sections of 4-μm thickness were prepared using a sliding microtome (SR-200; Leica), stained with HE and examined by light microscopy. Histochemical staining of multiple liver and kidney samples for neutral lipids was also performed. Sections previously fixed in 10% neutral formalin were cut using a cryotome (Cryotome FSE; Thermo Fisher Scientific, Runcorn, UK) and stained with Sudan IV according to a previously published method (10).

### Statistical analysis

All numerical data were analysed using Statistica 13.3 PL (TIBCO Software, Palo Alto, CA, USA). Data distribution was assessed for normality using the Shapiro–Wilk test, and homogeneity of variances was evaluated using Levene’s test. For variables meeting the assumptions of normality and equal variances, one-way ANOVA was applied, followed by Tukey’s honestly significant difference test for pairwise comparisons among the experimental groups (NC, PC, FR and BS). Variables that did not meet these assumptions were analysed using the non-parametric Kruskal–Wallis test, with Dunn’s *post-hoc* method being used for multiple comparisons. Results are presented as mean ± SD. Statistical significance was set at P-value ≤ 0.05, and highly significant differences were defined as P-value ≤ 0.01.

## Results

### Antioxidant and pro-oxidant markers

Significant differences in multiple antioxidant and pro-oxidant indices were observed in piglets from the FR and BS groups compared with piglets from the NC group (Table 1). Catalase activity was significantly higher in the FR and BS groups than in both control groups across plasma (P-value < 0.001), liver tissue (P-value = 0.003) and intestine tissue (P-value = 0.001), with the highest hepatic and intestinal values recorded in the BS group.

**Table 1.** Concentrations of antioxidant and pro-oxidant markers in plasma and hepatic and intestinal tissues of piglets

Marker	Positive control group	Negative control group	Fermented rapeseed meal group	*Bacillus subtilis* group	P-value	SEM
Plasma SOD (U/mL)	8.82 ± 0.537^ab^	6.10 ± 0.821^c^	8.51 ± 0.746^bc^	9.72 ± 0.255^a^	< 0.001	0.304
Plasma CAT (U/mL)	18.65 ± 1.87^b^	20.90 ± 0.926^b^	25.60 ± 1.78^a^	23.53 ± 0.710^a^	<0.001	0.611
Plasma FRAP (μmol/L)	10.66 ± 1.51^a^	8.21 ± 1.59^b^	11.10 ± 2.39^a^	10.81 ± 1.99^a^	<0.001	0.862
Plasma GSH (μmol/L)	0.640 ± 0.084^b^	0.679 ± 0.083^b^	0.709 ± 0.078^ab^	0.826 ± 0.060^a^	0.003	0.021
Plasma MDA (μmol/L)	3.65 ± 0.213^b^	4.66 ± 0.408^a^	2.27 ± 0.187^c^	2.16 ± 0.251^c^	<0.001	0.223
Plasma LOOH (μmol/L)	1.89 ± 0.064^a^	1.83 ± 0.093^ab^	1.75 ± 0.058^b^	1.74 ± 0.060^b^	0.001	0.019
Liver SOD (U/g)	74.15 ± 0.837^b^	78.55 ± 1.55^a^	79.18 ± 0.604^a^	77.06 ± 1.47^a^	<0.001	0.567
Liver CAT (U/g)	271.3 ± 34.79^bc^	259.5 ± 25.94^c^	323.4 ± 23.91^ab^	340.4 ± 21.71^a^	0.003	10.67
Liver GSH (μmol/g)	1.15 ± 0.076^b^	1.07 ± 0.097^b^	1.15 ± 0.097^b^	1.45 ± 0.068^a^	<0.001	0.042
Liver MDA (μmol/g)	3.74 ± 0.286^b^	4.40 ± 0.176^a^	3.31 ± 0.157^c^	3.21 ± 0.168^c^	<0.001	0.129
Liver LOOH (μmol/g)	1.33 ± 0.045^ab^	1.34 ± 0.017^a^	1.26 ± 0.040^b^	1.29 ± 0.021^ab^	0.027	0.011
Jejunum SOD (U/g)	61.66 ± 6.35^a^	56.91 ± 3.82^ab^	64.63 ± 4.15^a^	51.79 ± 4.55^b^	0.009	1.53
Jejunum CAT (U/g)	117.7 ± 11.39^b^	116.7 ± 30.85^b^	154.5 ± 38.89^b^	209.8 ± 27.10^a^	0.001	10.35
Jejunum GSH (μmol/g)	3.82 ± 0.358^a^	3.04 ± 0.164^b^	1.25 ± 0.065^c^	2.72 ± 0.157^b^	<0.001	0.237
Jejunum MDA (μmol/g)	1.41 ± 0.021^a^	1.35 ± 0.037^b^	1.30 ± 0.026^c^	1.28 ± 0.015^c^	<0.001	0.014
Jejunum LOOH (μmol/g)	1.94 ± 0.204^a^	1.23 ± 0.120^c^	1.56 ± 0.235^bc^	1.82 ± 0.198^ab^	<0.001	0.075

Positive control group – weaned piglets receiving a standard diet with feed additives and ZnO; negative control group – weaned piglets receiving the same diet as the positive control group but without feed additives or ZnO – fermented rapeseed meal group – weaned piglets receiving the same diet as the negative control group but with 8% content of this meal and *Bacillus subtilis* 87Y; *Bacillus subtilis* group – weaned piglets receiving the same diet as the negative control group with 0.1% *Bacillus subtilis* 87Y; SOD – superoxide dismutase; CAT – catalase; FRAP – ferric-reducing ability of plasma; GSH – glutathione; MDA – malondialdehyde; LOOH – lipid hydroperoxides;

a–c – different superscript letters in the same row indicate significant difference (P-value < 0.05). Data are least squares means for six replicate pens per diet and are shown as ±SD

Regarding GSH, significantly higher concentrations in plasma and liver were found in piglets from the BS group than in the NC group (P-value = 0.003 and P-value < 0.001, respectively). In the intestine, the FR group exhibited the lowest GSH values, the PC group the highest values, and the NC and BS groups intermediate values (P-value < 0.001).

A significant increase in SOD activity *versus* NC was observed in the plasma of piglets from the BS group (P-value < 0.001). In the liver, SOD activity was significantly lower in the PC group than in all other experimental groups (P-value < 0.001), whereas in the intestinal wall the lowest activity of this enzyme was recorded in the BS group and was significantly lower than activity in PC and FR (P-value = 0.009).

Lipid peroxidation products showed consistent patterns across all analysed tissues. In both the FR and BS groups, MDA concentrations were significantly lower in plasma, liver and intestine compared with NC (P-value < 0.001 for all comparisons). Significantly lower LOOH concentrations in the liver were observed in the FR group than in the NC group (P-value = 0.027), and significantly lower ones were recorded in the intestinal wall in the FR group than in the PC group (P-value < 0.001). In plasma the lowest LOOH concentration was observed in piglets from the BS group, differing significantly from that in the PC group piglets (P-value = 0.001). In plasma the lowest LOOH concentration was observed in piglets from the BS group, differing significantly from that in the PC group piglets (P-value = 0.001) (Table 1).

### Biochemical and enzyme parameters

Significant between-group differences were observed for selected plasma biochemical indices in piglets (Table 2). Plasma Glu concentration was significantly lower in the BS group compared with all other experimental groups (P-value < 0.001). Significantly higher TP and GLOB were noted in the FR and BS groups than in the PC and NC groups (P-values < 0.001 and 0.047, respectively). In contrast, ALB did not differ significantly between groups (P-value = 0.060).

**Table 2.** Biochemical and enzymatic parameters in piglet plasma

Biomarker/enzyme	Positive control group	Negative control group	Fermented rapeseed meal group	*Bacillus subtilis* group	P-value	SEM
Glu (mmol/L)	5.58 ± 0.291^a^	5.47 ± 0.260^a^	5.35 ± 0.257^a^	4.61 ± 0.286^b^	<0.001	0.095
TP (g/L)	64.02 ± 3.10^b^	63.13 ± 6.09^b^	72.08 ± 2.26^a^	73.93 ± 2.70^a^	<0.001	1.24
ALB (g/L)	29.52 ± 3.07	29.85 ± 3.60	32.70 ± 2.33	34.08 ± 3.59	0.060	0.728
GLOB (g/L)	34.50 ± 2.69^b^	33.28 ± 5.73^b^	39.38 ± 2.25^a^	39.85 ± 5.78^a^	0.047	1.04
ALB/GLOB	0.863 ± 0.146	0.919 ± 0.191	0.834 ± 0.094	0.877 ± 0.189	0.831	0.031
Urea (mmol/L)	5.11 ± 0.653^a^	3.61 ± 0.087^b^	3.55 ± 0.190^b^	5.16 ± 0.717^a^	<0.001	0.188
CREAT (μmol/L)	74.26 ± 6.87^b^	85.16 ± 7.04^a^	69.69 ± 4.35^b^	75.73 ± 6.91^ab^	0.004	1.69
BIL (μmol/L)	2.88 ± 0.768	2.59 ± 0.070	2.62 ± 0.140	2.59 ± 0.070	0.539	0.079
CHOL (mmol/L)	1.87 ± 0.100	1.89 ± 0.076	1.85 ± 0.097	1.78 ± 0.151	0.348	0.023
HDL (mmol/L)	1.15 ± 0.102^b^	1.15 ± 0.072^b^	1.31 ± 0.090^a^	1.24 ± 0.132^ab^	0.037	0.024
LDL (mmol/L)	0.571 ± 0.082^a^	0.582 ± 0.091^a^	0.408 ± 0.098^b^	0.415 ± 0.035^b^	0.001	0.023
% HDL	61.42 ± 3.79^b^	60.68 ± 3.80^b^	70.72 ± 3.89^a^	69.60 ± 2.61^a^	<0.001	1.171
HDL:CHOL	0.614 ± 0.038^b^	0.607 ± 0.022^b^	0.707 ± 0.027^a^	0.696 ± 0.026^a^	<0.001	0.012
TG (mmol/L)	0.332 ± 0.047^ab^	0.355 ± 0.058^a^	0.294 ± 0.048^ab^	0.273 ± 0.034^b^	0.030	0.011
ALT (U/L)	28.58 ± 2.96^b^	41.03 ± 1.88^a^	37.78 ± 1.16^a^	41.10 ± 1.80^a^	<0.001	1.14
AST (U/L)	46.27 ± 2.32^bc^	44.32 ± 3.15^c^	49.73 ± 1.81^ab^	51.17 ± 3.59^a^	0.001	0.778
LDH (U/L)	649.8 ± 64.80	687.3 ± 83.60	727.2 ± 54.35	680.3 ± 57.61	0.275	13.83
CK (U/L)	1,253.3 ± 101.6^b^	1,589.0 ± 140.07^a^	528.2 ± 50.87^d^	708.2 ± 52.52^c^	<0.001	90.09
GGTP (U/L)	18.44 ± 1.35^b^	33.33 ± 3.31^a^	29.64 ± 3.42^a^	32.24 ± 2.41^a^	<0.001	1.34

Positive control group – weaned piglets receiving a standard diet with feed additives and ZnO; negative control group – weaned piglets receiving the same diet as the positive control group but without feed additives or ZnO – fermented rapeseed meal group – weaned piglets receiving the same diet as the negative control group but with 8% content of this meal and *Bacillus subtilis* 87Y; *Bacillus subtilis* group – weaned piglets receiving the same diet as the negative control group with 0.1% *Bacillus subtilis* 87Y; Glu – glucose; TP – total protein; ALB – albumin; GLOB – globulins; CREAT – creatinine; BIL – bilirubin; CHOL – total cholesterol; HDL – high-density lipoprotein cholesterol; LDL – low-density lipoprotein cholesterol; TG – triacylglycerols; ALT – alanine aminotransferase; AST – aspartate aminotransferase; LDH – lactate dehydrogenase; CK – creatine kinase; GGTP – gamma-glutamyltransferase;

a–d – different superscript letters in the same row indicate significant difference (P-value < 0.05). Data are least squares means for six replicate pens per diet and are shown as ±SD

Regarding nitrogen-related indices, urea concentrations were higher in PC and BS than in NC and FR (P-value < 0.001), whereas CREAT concentrations were significantly higher in NC than in PC and FR (P-value = 0.004).

Analysis of plasma lipid indices in piglets revealed significant between-group differences for selected parameters (Table 2). High-density lipoprotein cholesterol concentration was significantly higher in the FR group than in the two control groups (P-value = 0.037), which was also reflected in a higher proportion of HDL (%HDL) (P-value < 0.001) and a higher HDL : CHOL ratio (P-value < 0.001). An opposite pattern was observed for LDL, the plasma concentration of which was significantly lower in the FR and BS groups than in the PC and NC groups (P-value = 0.001). The TG concentration was significantly lower in the BS group than in the NC group (P-value = 0.030).

Analysis of enzyme activities in piglet plasma showed a significant increase in ALT activity in the NC, FR and BS groups compared with the PC group (P-value < 0.001). Plasma AST activity reached significantly higher values in the BS and FR groups than in the NC group (P-value = 0.001). Creatine kinase activity differed significantly among groups, following the pattern FR < BS < PC < NC (P-value < 0.001), whereas GGTP activity was significantly higher in the NC, FR, and BS groups than in the PC group (P-value < 0.001) (Table 2).

### Organ weights

Analysis of visceral organ weights revealed significant between-group differences only for the liver (P-value = 0.002). Liver weight was lower in the BS group compared with the PC and NC groups. No significant differences were observed for heart or kidney weights (P-value = 0.592 and P-value = 0.135, respectively) (Table 3).

### Histopathological findings

Microscopic examination of multiple liver samples revealed no marked differences in liver structure between animals from the different experimental groups. The liver parenchyma was divided into lobules separated by thin bands of connective tissue. In individual lobules, hepatocyte trabeculae were regularly arranged and radiated towards the central vein. Hepatocytes usually contained a single centrally located nucleus, with occasional binucleated cells. Basophilic granules were evenly distributed throughout the cytoplasm. Browicz–Kupffer cells were present in moderate numbers between the hepatocyte trabeculae. In the peripheral zone of the lobules, portal triads containing branches of the hepatic artery and portal vein were visible, as well as bile ducts or their branches (Fig. 2).

**Fig. 2.**
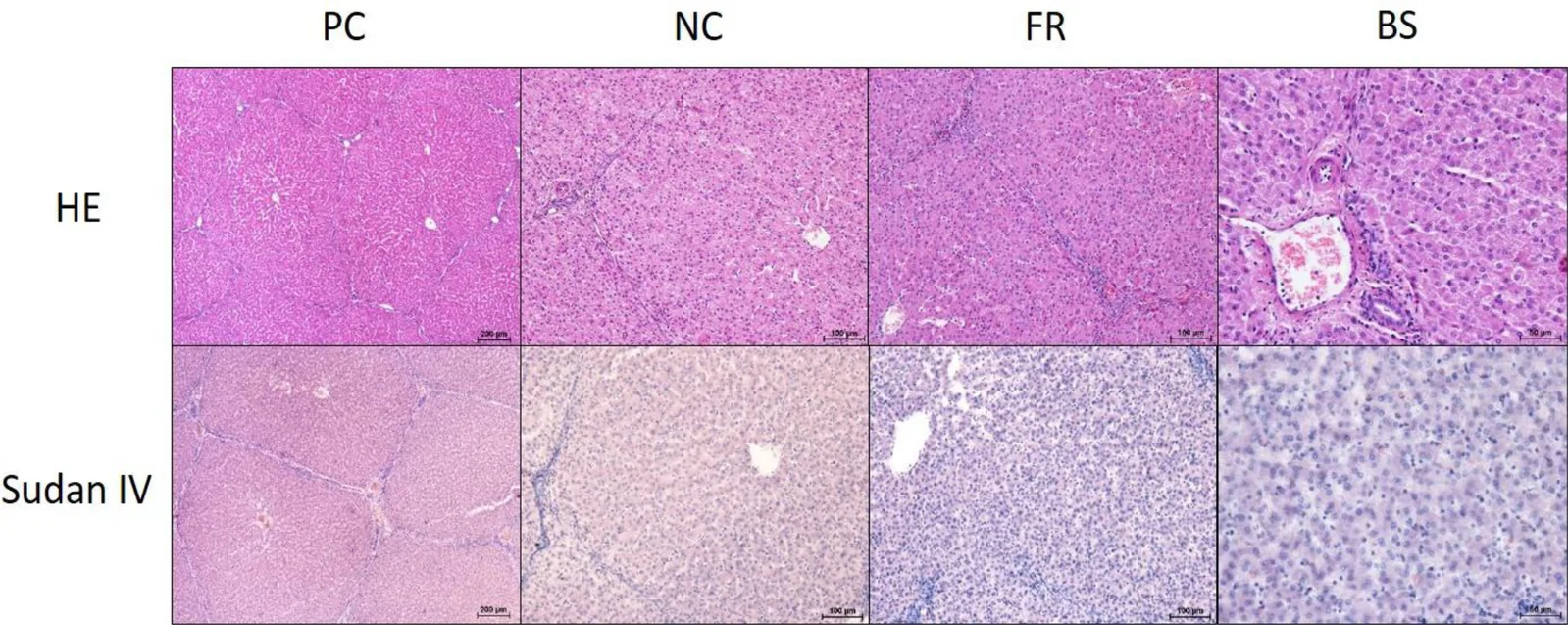
Representative histological images of liver tissue from piglets in the PC, NC, FR and BS groups. Staining with HE shows liver parenchyma divided into hepatic lobules with visible central veins and portal triads separated by thin bands of connective tissue. Staining with Sudan IV dye shows very weak histochemical staining for neutral lipids, visible as small orange lipid vacuoles in the cytoplasm of individual hepatocytes. PC – positive control group of weaned piglets receiving a standard diet with feed additives and ZnO; NC – negative control group of weaned piglets receiving the same diet as the positive control group but without feed additives or ZnO; FR – fermented rapeseed meal group of weaned piglets receiving the same diet as the negative control group but with 8% content of this meal and *Bacillus subtilis* 87Y; BS – *Bacillus subtilis* 87Y group of weaned piglets receiving the same diet as the negative control group with 0.1% *Bacillus subtilis* 87Y

Sudan IV staining revealed only negligible amounts of neutral lipids, visible as very small vacuoles in individual hepatocytes. No irreversible cellular changes were observed. No evident differences in neutral lipid accumulation were found between the experimental groups (Fig. 2).

Similarly to the liver, kidney samples also revealed no marked structural differences between animals from the different experimental groups under microscopic examination. A normal number of glomeruli with preserved filtration spaces were observed, evenly distributed throughout the renal cortex. In cross-sections of the renal cortex, proximal and distal renal tubules lined with simple cuboidal epithelium were also visible. The renal medulla, containing nephron loops, consisted mainly of cross-sections of collecting tubules and papillary ducts, with sparse stromal connective tissue between them (Fig. 3). Staining with Sudan IV dye revealed only minimal lipid deposits in the tubular epithelium, visible as small lipid vacuoles. No marked differences in lipid accumulation or irreversible cellular changes were observed between the experimental groups (Fig. 3).

**Fig. 3.**
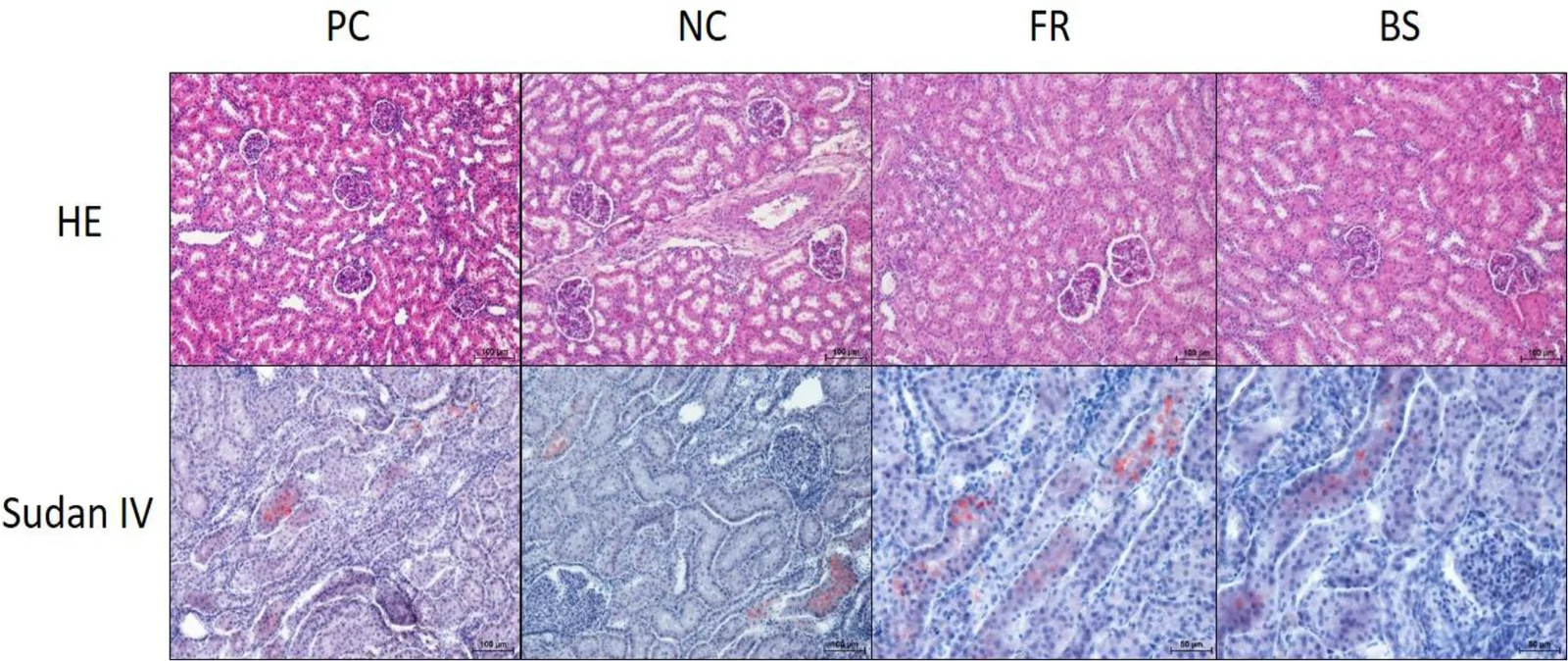
Representative histological images of kidney tissue from piglets in the PC, NC, FR and BS groups. Staining with HE shows glomeruli with preserved filtration spaces and adjacent proximal and distal convoluted tubules lined with simple cuboidal epithelium. Staining with Sudan IV dye shows scattered, tiny lipid vacuoles in the cytoplasm of the renal tubular epithelium. PC – positive control group of weaned piglets receiving a standard diet with feed additives and ZnO; NC – negative control group of weaned piglets receiving the same diet as in the positive control group but without feed additives or ZnO; FR – fermented rapeseed meal group of weaned piglets receiving a diet with 8% content of this meal and *Bacillus subtilis* 87Y without feed additives or ZnO; BS – *Bacillus subtilis* 87Y group of weaned piglets receiving a diet with 0.1% *Bacillus subtilis* 87Y without feed additives or ZnO

## Discussion

Probiotics are widely used in piglet nutrition to stimulate immunity and support antioxidant mechanisms, of which the activity during weaning is limited and prone to redox imbalance (23, 34). Most commonly, strains of *Lactobacillus, Enterococcus, Bifidobacterium, Saccharomyces* (probiotic yeasts) and certain *Streptococcus* strains are applied; however, *Bacillus* sp. are gaining importance owing to their stability, ability to form endospores, and broad spectrum of potential health-promoting effects (1, 30). The newly isolated *Bacillus subtilis* 87Y strain, also used by the present researchers previously in the fermentation of rapeseed meal, has been shown to support redox balance and overall health status in piglets (10, 33). In the present study, the effects of *Bacillus* sp. 87Y on oxidative and metabolic status in weaned piglets were evaluated. The strain’s effects were compared between two delivery matrices, namely direct supplementation as spores and fermented rapeseed meal containing *Bacillus* sp. 87Y, as well as against two control groups, those being a negative control receiving a basal diet without additives and a positive control fed a standard diet containing ZnO as routinely used to support piglet health. This design enabled assessment not only of which delivery matrix for *Bacillus* sp. 87Y (spores *versus* fermented carrier) was more effective, but also whether *Bacillus subtilis* 87Y could serve as an alternative to ZnO as a component supporting immunity and redox homeostasis in piglets.

The findings indicate that both *Bacillus subtilis* 87Y supplementation and inclusion of rapeseed meal fermented with *Bacillus subtilis* 87Y improved oxidative status during weaning compared with the additive-free control. Both delivery forms modified antioxidant-related indices in young piglets, including higher CAT activity and compartment-dependent changes in SOD and GSH. In this context, not only traditional molecules such as GSH and indices of overall FRAP should be considered, but also selected plasma biochemical parameters including urea; liver enzymes (ALT, AST and GGTP); and BIL, TP and GLOB. These variables together provide complementary information on oxidative status, detoxification processes, and the maintenance of redox homeostasis (12).

The concurrent reduction in lipid peroxidation products such as MDA and LOOH further indicates that both probiotic delivery forms were effective in limiting oxidative stress, one of the key factors constraining health and growth during the rapid development of young pigs (16). The beneficial effects observed with direct *Bacillus subtilis* 87Y administration (in the BS group) are likely related to stimulation of endogenous antioxidant defences. The probiotic may also contribute to host health by modulating gut microbiota composition and function and by limiting the translocation of potentially pathogenic microorganisms into the bloodstream (5).

Comparable mechanisms have been reported for other probiotic strains. For example, supplementation with *Bacillus velezensis* improved intestinal antioxidant capacity in piglets, alleviated diarrhoea, and enhanced production performance by influencing gut microbiota balance and the expression of immunity-related genes (21). High-dose supplementation with *Bacillus subtilis* M-1 improved growth indices and reduced oxidative stress markers in piglets; the strain also strengthened intestinal barrier function and modulated immune responses, although some impairment of immunity was reported (31). In addition, *Lactobacillus johnsonii* RS-7 improved the antioxidant status of weaned piglets by increasing antioxidant enzyme activity and enhancing immune functions (35).

Notably, the effects of *Bacillus subtilis* 87Y either as spores or in fermented rapeseed meal were also evident at the organ level. In the BS group, a lower liver weight was observed, which, together with histological findings, suggests a reduced metabolic burden and improved protection against oxidative stress. Microscopic observations indicated better hepatocyte structure in the BS and FR groups, with less pronounced degenerative changes, vacuolisation and necrotic foci compared with the NC group (although these alterations were mild). It is also worth noting that livers from piglets in the PC group, despite ZnO supplementation, showed slightly higher degrees of structural changes than those in the probiotic-fed groups. This suggests that *Bacillus subtilis* 87Y may exert tissue-level protective effects by stabilising redox processes and limiting lipid membrane damage. Similar hepatoprotective effects of probiotics in pigs have been reported by Sarkar *et al*. (27) and Zhao *et al*. (35), indicating that improved redox balance translates into a more favourable histological profile of metabolic organs.

Extending the interpretation of *Bacillus subtilis* 87Y effects to the biochemical outcomes, probiotic supplementation either as spores or *via* fermented feed appeared to influence protein and carbohydrate metabolism, as reflected by increased TP and GLOB and modulation of the ALB : GLOB ratio. Changes in urea concentration and in the liver enzymes ALT, AST and GGTP suggest that probiotic additives may support hepatic detoxification and nitrogen metabolism, consistent with previous reports showing improved liver function in piglets supplemented with *Bacillus* or *Lactobacillus* strains (22). Moreover, Deng *et al*. (13) reported that inclusion of *Bacillus subtilis* in the diet supported lipid, protein and carbohydrate metabolism and promoted maturation of the intestinal mucosa in piglets, in line with observations by Huo *et al*. (20). The reduction in CK activity in the FR and BS groups may additionally indicate attenuation of oxidative damage to skeletal muscle and the heart.

The observed modulation of the lipid profile, including reduced LDL and increased HDL in the plasma of piglets receiving diets containing *Bacillus subtilis* 87Y, indicates a close association between oxidative stress reduction and improved lipid metabolism. Decreased lipid peroxidation of membrane lipids favours stabilisation of plasma and tissue lipid fractions, enhancing lipid transport efficiency, lipoprotein synthesis and secretion, and maintaining the hepatocyte and enterocyte membrane structural integrity crucial for proper lipid metabolism and redistribution (2). In the FR group, an additional effect likely resulted from the presence of bioactive fermentation metabolites, including lipopeptides, lactic acid and readily digestible nutrients. These compounds may modulate antioxidant enzyme activity, improve tissue redox homeostasis, and increase lipid bioavailability by optimising intestinal barrier function and the intestinal microenvironment (10). Consequently, the synergistic effects of *Bacillus subtilis* 87Y and fermentation metabolites support a favourable lipoprotein profile, protect hepatocytes against oxidative stress, and stabilise metabolic functions during the peri-weaning period (27). This integrated action suggests that administration of this probiotic strain in fermented rapeseed meal improves not only antioxidant status but also lipid metabolism, which is essential for optimal growth and health of piglets during this rearing phase when they are vulnerable. Similar mechanisms have been demonstrated for other probiotic strains, such as *Bacillus velezensis* and *Lactobacillus johnsonii* RS-7, which stimulate antioxidant gene expression, improve lipid profiles, and modulate intestinal immune functions in young pigs (21). In light of the available literature presenting beneficial effects of fermented rapeseed meal on piglet health, the effects observed in the FR group suggest that fermented rapeseed meal acted as a bioactive carrier, increasing nutrient bioavailability and enhancing the potential of *Bacillus subtilis* 87Y to improve both redox processes and lipid metabolism, which is critical for maintaining metabolic homeostasis during rapid growth (9, 10, 33).

Importantly, the effects achieved in the BS and FR groups were comparable, and in some indices even better than those observed in the PC group receiving the permitted level of ZnO. The probiotic diets clearly outperformed the NC group’s diet lacking both ZnO and probiotic/prebiotic additives. These observations suggest that a properly selected probiotic or fermented component may effectively reduce the need for ZnO as the traditional strategy aimed at supporting immunity and affording protection against oxidative stress during weaning.

## Conclusion

The research comprehensively evaluated the efficacy of the novel *Bacillus subtilis* 87Y strain administered to piglets in both pure form and in a fermented matrix. The obtained results clearly indicate that supplementation with this strain, regardless of the delivery form applied, led to significant improvement in the redox status in piglet organisms. A distinct increase in the activity of key antioxidant enzymes (CAT and SOD) and an elevation in GSH concentration were noted, accompanied by a simultaneous reduction in the levels of lipid peroxidation markers (MDA and LOOH) in plasma and in liver and intestine tissue. These changes were accompanied by improvements in lipid metabolism parameters and beneficial modifications of liver enzyme activities, which collectively suggest a hepatoprotective effect and stabilisation of metabolic balance during the peri-weaning period. Comparison of the delivery forms revealed that *B. subtilis* 87Y in the form of a pure probiotic did not yield inferior efficacy to the fermented variant. Comparison of *B. subtilis* 87Y and ZnO indicated that in selected parameters, the bacterium may even surpass the effect of the trace mineral supplement.

The conducted analyses demonstrate the strain *B. subtilis* 87Y to have high utility as an innovative feed additive supporting health, oxidative resilience and liver function in piglets, with its efficacy remaining largely independent of the applied delivery matrix.
